# Time to achieve optimal glycemic control and its determinants among diabetes mellitus patients receiving treatment: a retrospective study

**DOI:** 10.1038/s41598-025-96097-1

**Published:** 2025-06-06

**Authors:** Maru Zewdu Kassie, Chekol Alemu, Habitamu Wudu, Buzuneh Tasfa Marine, Asaye Alamneh Gebeyehu

**Affiliations:** 1 Department of Statistics, College of Natural and Computational Sciences, Assosa University, Assosa, Ethiopia; 2 Department of Statistics, College of Natural and Computational Sciences, Gambella University, Gambella, Ethiopia; 3 Department of Epidemiology, Faculty of Public Health, Jimma University, Jimma, Ethiopia; 4 Department of Public Health, College of Health Sciences, Debre Tabor University, Debre Tabor, Ethiopia

**Keywords:** Diabetes mellitus, Time to optimal glycemic control, Cox-PH model, Retrospective study, And blood glucose level, Biomarkers, Diseases, Endocrinology, Medical research, Risk factors

## Abstract

Diabetes mellitus (DM) is a major public health problem responsible for morbidity and mortality. Maintaining blood sugar control helps patients achieve optimal glycemic levels. Therefore, this study aimed to identify the factors affecting the time to achieve optimal glycemic control among DM patients at Assosa General Hospital (AGH), Western Ethiopia. A retrospective study design was conducted from 427 randomly selected DM patients in the outpatient department (OPD) clinic at AGH under the follow-up period from September 2022 to September 2024. The median survival time, Kaplan-Meier survival estimate, and Log-Rank test were used to describe the data and compare the survival time between groups. The study used Cox PH model to analyze the time to achieve optimal glycemic control of DM patients, where hazard ratio, p-value, and 95% CI for hazard ratio were used for testing significance. Schoenfeld and Cox-Snell residuals were used to check the model assumptions. The median time to optimal glycemic control for DM patients was 12 months. At the end of the follow-up, 74.2% of the patients had developed an event and the rest 25.8% were censored. The significant predictors of time to optimal glycemic control include: older age (AHR = 0.871(95% CI 0.809, 0.937)), females (AHR = 1.295 (95% CI 1.024, 1.639)), having FHDM (AHR = 1.681(95% CI 1.313, 2.153)), rural residence(AHR = 0.463(95% CI 0.354, 0.607)), presence of comorbidity (AHR = 0.508(95% CI 0.302, 0.854)), DM related complications (AHR = 0.419(95% CI 0.326, 0.539)), high BLBGL AHR = 0.997(95% CI 0.995, 0.998)). This study found the factors that prolonged or shortened the time to reach optimal glycaemic control for T2DM patients. The study revealed that older age, male patients, patients having other related comorbidities and patients with no FHDM, patients having DM-related complications as poor prognostic factors of T2DM disease and also prolonged recovery time. Therefore, attention should be given to these patients to obtain good glycaemic levels and the patient being healthy.

## Introduction

Diabetes mellitus is a disease in which the body does not produce or respond properly to insulin^1,2^. It is a lifetime challenge that requires proper self-care practices for better glycemic control^3^. Although T1DM is also increased rapidly with many complications, T2DM is a highly distributed and serious health problem in the world^4,5^. This problem is also critical in Ethiopia specifically in the study area^6,7^. In 2019, it is estimated that more than 463 million people were living with diabetes worldwide and this is expected to reach about 578 million and 700 million by 2030 and 2045 respectively^8^. For a long time, Africa was considered safe from many of the diseases that are called “diseases of affluence,” like diabetes mellitus which can affect the developed countries like the Western world^8,9^. However, now it is a common problem in Africa specifically in Ethiopia^9^. The previous studies^10–12^ indicated that from the common reason for a high distribution of diabetes mellitus in people, urbanization plays a vital role and needs further investigation of why this could happen.

In Ethiopia, diabetic patients must travel long distances to obtain insulin and tablets since there is scarce of health facilities^13^. Insulin is one of the most valuable anti-diabetic medications that control the patient blood sugar level^14^. When DM patients follow their medication properly, the disease progression and blood sugar level decrease spontaneously, and also their beta cells work effectively as a result they tend to decrease their insulin requirements as compared to prior doses^15,16^. This period is referred to as a honeymoon or remission period^17,18^. If diabetic patients manage their blood sugar levels through integrated self-care and professional health support, they can live longer and lead healthier lives^15,16^. Therefore, controlling blood sugar is crucial for preventing and slowing the progression of complications^19,20^.

Searching for factors affecting the time to optimal glycemic control is crucially important, as disease severity, mortality, and recovery vary among individuals due to various factors. A study^21^ revealed that patients with diabetes-related complications and other comorbidities, such as HIV/AIDS, kidney disease, stroke, and hypertension, have a lower likelihood of achieving glycemic control. Another study showed that older age and the presence of pre-existing comorbidities delayed recovery time from diabetes mellitus^22,23^. Additionally, a previous study indicated that female diabetic patients recover faster than male patients and those with less or no education controlled the disease better than educated individuals^24^. Conversely, another study found that males had a higher probability of achieving target diabetes control^25^, while other studies reported that educated patients managed the disease better than their less-educated counterparts^26,27^. Hence, there are inconsistencies in the findings of previous studies, necessitating further investigation.

Previous studies have been conducted on the survival analysis of diabetes mellitus, some of which considered cross-sectional cases, particularly research on the time to recovery from diabetes mellitus^28–31^. However, these researches used the Cox-PH (Cox Proportional Hazard) model without checking the proportional hazard assumption. This assumption is a precondition to using this model. If this assumption is violated, the simple Cox model is invalid, and more sophisticated analysis; parametric accelerated failure time models are required^32–34^. In our study, we have addressed these issues by checking the assumption using the Schoenfeld residual plot and its standard statistical tests. Additionally, conducting several studies in different settings is very important to get reasonable and authentic information. While previous studies have investigated glycemic control in different settings, limited research has focused on Western Ethiopia, specifically Assosa General Hospital. This study provides localized evidence that can inform region-specific healthcare interventions. This study, therefore, aims to identify the determinants of the time to reach optimal glycemic control among T2DM patients under treatment at Assosa General Hospital, Assosa, Ethiopia.

## Methods

### Study area and study design

The study was conducted at Assosa General Hospital (AGH), a major public healthcare facility located in Western Ethiopia. As the principal referral hospital in the Benishangul-Gumuz region, AGH plays a critical role in delivering both curative and preventive healthcare services to an estimated more than 200,000 patients annually from the region and neighboring districts. The hospital is staffed by approximately 520 healthcare professionals, including doctors, nurses, specialists, and other administrative workers ensuring the provision of comprehensive medical services across various disciplines.

AGH is well-equipped to manage a broad spectrum of health conditions, with a strong emphasis on chronic disease management. The hospital provides specialized care for non-communicable diseases such as hypertension, diabetes mellitus, and cardiovascular disorders. Its diabetes care services include routine follow-ups, medication management, lifestyle counseling, early complication screening, and patient education programs aimed at improving self-management and disease prevention. Additionally, AGH has well-established outpatient and inpatient services, laboratory and diagnostic facilities, and a multidisciplinary team working to enhance the quality of healthcare delivery. For this study, a retrospective study design was employed to systematically retrieve relevant information from the medical records of diabetes mellitus patients.

### Study population and study period

The study population consisted of T2DM patients who were under follow-up for insulin medication at AGH and initiated treatment between September 2022 and September 2024. The diagnosis of T2DM was made based on the American Diabetes Association (ADA) criteria, ensuring that all included patients met internationally recognized diagnostic standards.

During the study period, a total of 1,016 T2DM patients were registered at AGH. However, only 427 patients met the inclusion criteria and were included in the final analysis. These patients were followed until they either achieved optimal glycemic control (event) or were censored.

### Source of data and data collection procedures

The primary source of data for this study was the medical records of T2DM patients. Data collection involved reviewing individual patient charts and follow-up cards in the outpatient department (OPD) at AGH for the specified follow-up period from September 2022 to September 2024. The data collection process was conducted by a trained team consisting of four professional nurses and one statistician.

Patients were followed up at regular intervals of every 1 to 3 months, depending on their clinical status, medication adherence, and blood glucose levels. Each follow-up visit included clinical evaluations, laboratory investigations, medication adjustments, and lifestyle counseling. The time to reach optimal glycemic control (event time variable) was recorded as the time from treatment initiation to the first achievement of normal blood sugar levels. A patient was considered to have achieved glycemic control when their blood sugar level fell within the normal range (70–130 mg/dl) or by long-term measure HbA1c < 7.5%, with targets adjusted based on factors such as age, comorbidities, and other complications^14,35^.

The patient charts used for data collection were standardized forms developed by the Federal Ministry of Health, ensuring consistency in documentation and facilitating the early identification and tracking of clinical and laboratory measurements. This study exclusively relied on secondary data obtained from these follow-up charts.

### Inclusion and exclusion criteria

Inclusion Criteria:

- T2DM patients aged 18 years and above who were diagnosed based on ADA criteria.
- Patients who initiated insulin treatment at AGH during the study period (September 2022 – September 2024).
- Patients who had at least three follow-up visits during the study period.

Exclusion Criteria:

- Patients younger than 18 years.
- Patients with fewer than three follow-up visits.
- Patients whose registration and treatment initiation did not fall within the study period.
- Patients with incomplete medical records, making it impossible to track their follow-up status.

**Fig. 1:**
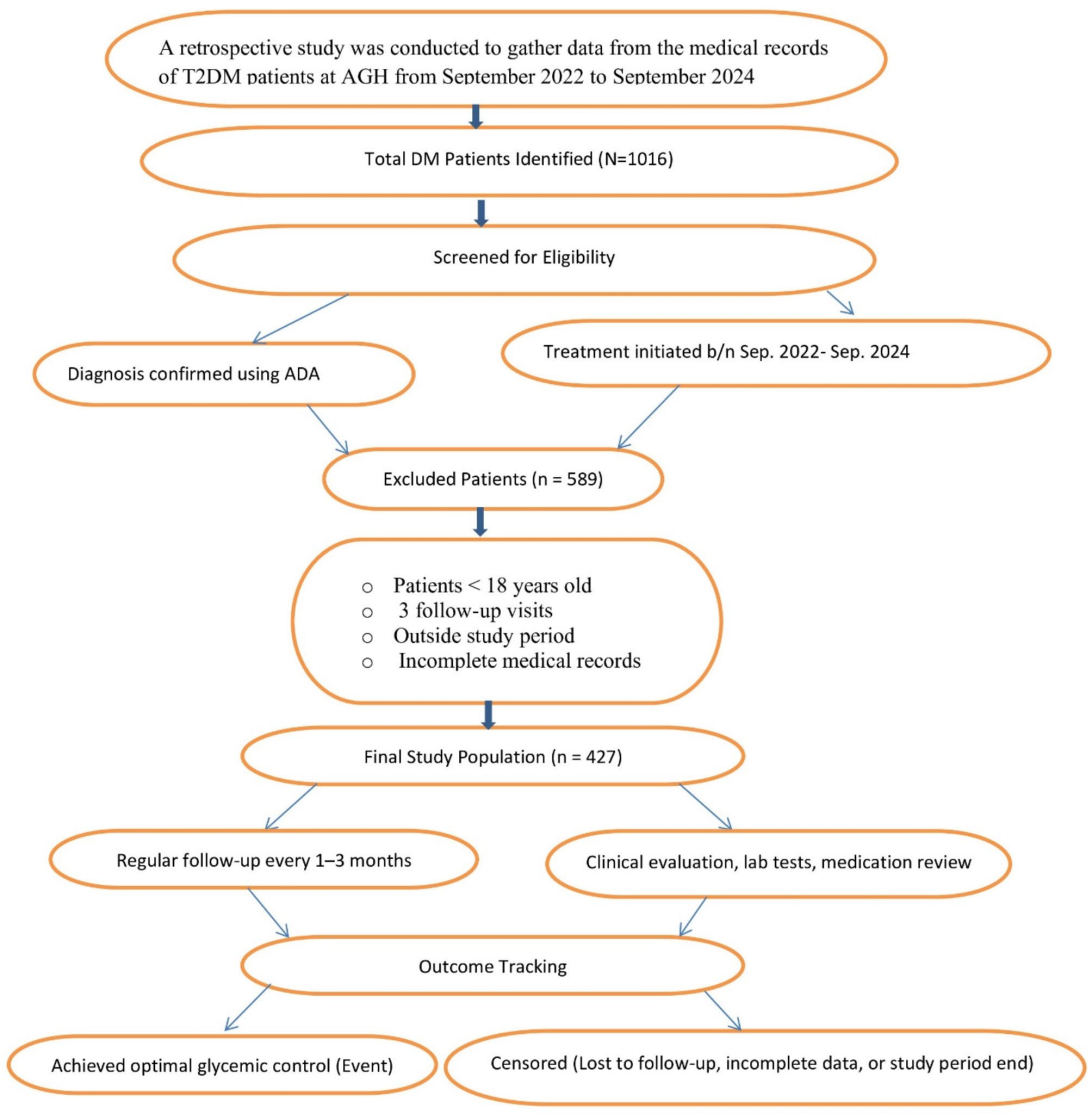
Patient enrollment flowchart for T2DM study at Assosa General Hospital.

To enhance clarity and facilitate a better understanding of the patient selection process, look at a detailed patient enrollment flowchart in (Fig. 1). This flowchart visually represents the inclusion, exclusion, and follow-up process of study participants.

### Operational definitions

**Time to optimal glycemic control**: According to the American Diabetes Association (ADA), International Diabetes Federation (IDF), and other clinical guidelines, a patient was considered to have achieved glycemic control when their fasting blood sugar level fell within the normal range of 70–130 mg/dL, or when their long-term measure, HbA1c, was < 7.5%. Targets may be adjusted based on factors such as age, comorbidities, and other complications^14,35^.

**Right censoring**: is considered when the patient is not recovered once between the study time, is transferred to another hospital, and died before reaching optimal glycemic control.

**Comorbidity** was defined as the presence of at least one other chronic condition other than diabetes mellitus, such as a physical non-communicable disease, a mental health condition, or an infectious disease^36,37^.

**DM-related complications** refer to health problems that arise as a result of prolonged or poorly controlled DM.

### Study variables

#### Response variable

The response variable in this study was the time to achieve optimal glycemic control for T2DM patients, defined as the time from the start of treatment until the first normal blood sugar level was reached during the follow-up period.

#### Explanatory variables

The study considers the following potential explanatory variables: Age in years, Gender (male, female), Place of residence (rural, urban), Presence of comorbidity (no, yes), Family history of DM (no, yes), presence of Hypertension (no, yes), Presence of CKD, presence of DM related complications (no, yes), weight in kg and Hemoglobin in g/dl.

### Method of data analysis

In this study, the Cox proportional hazards model was employed to assess the time to optimal glycemic control among T2DM patients. This model is widely used in survival analysis due to its flexibility and ability to handle censored data. Data were analyzed using R software version 4.1.1 and SPSS version 27, ensuring robust statistical computation and model validation.

### Descriptive statistics

Descriptive statistics were used to summarize the study variables. Frequency tables were constructed to present categorical data distributions, while median survival times were estimated to describe the central tendency of time-to-event outcomes. The Kaplan-Meier estimator was used to generate survival curves, allowing for a non-parametric visualization of the time-to-optimal glycemic control across different predictor categories. The log-rank test was conducted to compare survival distributions between groups.

### Survival data analysis

Survival analysis is a statistical approach designed to analyze time-to-event data while accounting for censoring. The **Cox proportional hazards (PH) regression model** was applied due to its semi-parametric nature, which does not require assumptions about the baseline hazard function. The hazard function at time $\:t$ is expressed as:

$$\:h\left(t,{x}_{i},\beta\:\right)=\:{h}_{0}\left(t\right)\text{e}\text{x}\text{p}\left({x}_{i}^{T}\beta\:\right)$$

where, $\:{h}_{0}\left(t\right)$ is the baseline hazard function; $\:{x}_{i}$ is a vector of covariates and $\:\beta\:$ is a vector of parameter estimates. Note that; $\:{h}_{0}\left(t\right)$ is the hazard function, where all values of the covariates are zero ($\:\text{exp}\left({x}_{i}^{T}\beta\:\right)=1$). Parameter estimate $\:\beta\:$ refers to the increase in log hazard with a one-unit increase for the continuous covariate. The proportional hazards assumption (i.e., the ratio of hazards remains constant over time) was rigorously tested using the Schoenfeld residual test to verify model validity^32,34^. Additionally, Cox-Snell residuals, Schoenfeld residuals, deviance residuals, and Dfbeta values were examined to assess the model’s goodness of fit, proportionality assumption, and potential influential observations^38,39^. These diagnostic checks ensured the reliability and scientific rigor of the survival model in estimating the effects of covariates on time to optimal glycemic control.

## Results

Among the 427 T2DM patients included in the study, the distribution of gender, residence, family history of diabetes mellitus (FHDM), presence of other related diseases, and hypertension is analyzed in relation to observed events (achievement of glycemic control) and censored cases (those who did not achieve glycemic control during the study period).

Regarding gender, females constituted 52.2% of the total participants, while males made up 47.8%. Among females, 44.7% achieved optimal glycemic control (observed events), while 7.5% were censored. For males, 29.5% achieved glycemic control, with 18.3% censored. This indicates that females had a higher proportion of observed events compared to males, suggesting potential gender differences in treatment outcomes or adherence.

In terms of residence, 55% of the patients were from rural areas, while 45% were from urban areas. Among rural residents, 43.8% achieved glycemic control, while 11.2% were censored. In urban residents, 30.4% achieved glycemic control, and 14.5% were censored. The data shows a higher success rate in rural areas, which may be attributed to differences in lifestyle, diet, or other factors.

For family history of diabetes mellitus (FHDM), 63.0% of the patients had no family history, while 37.0% reported a positive family history. Among those without a family history, 45.7% achieved glycemic control, while 17.3% were censored. Among those with a family history, 28.6% achieved glycemic control, and 8.4% were censored. This suggests that patients with a family history of diabetes may take longer to achieve glycemic control or face more challenges in managing their condition.

Regarding comorbidity, 71.2% of the participants had no DM-related comorbidity, while 28.8% reported having at least one related comorbid condition. Among those without related comorbidities, 55.5% achieved glycemic control, while 15.7% were censored. For those with related comorbidities, 18.7% achieved glycemic control, and 10.1% were censored. The presence of other related comorbidities appears to hinder achieving optimal glycemic control.

In the case of hypertension, 78.5% of the participants did not have hypertension, while 21.5% had hypertension. Among those without hypertension, 57.8% achieved glycemic control, while 20.6% were censored. Among those with hypertension, 16.4% achieved glycemic control, and 5.2% were censored. This indicates that hypertension may negatively affect glycemic control outcomes.

The study also examined the impact of a history of diabetes-related complications and comorbidities. Among the participants, 22.2% had a history of diabetes-related complications, while 77.8% did not. Patients without complications exhibited a higher proportion of events (59.3%) compared to those with complications (15.0%). Regarding comorbidities, 71.2% of the patients had no comorbid conditions, while 28.8% had one or more comorbidities. Those without comorbidities had a greater proportion of events (55.5%) compared to those with comorbidities (18.7%), indicating that comorbid conditions may hinder achieving optimal glycemic control.

The baseline continuous covariates of the participants were analyzed to provide an overview of their initial clinical characteristics. The mean weight of the participants was 58.26 kg, with a standard deviation of 4.93 kg. The average age of the participants was 43.85 years, with a standard deviation of 3.74 years, indicating a wide variation in age among the study population. The mean hemoglobin level was found to be 12.76 g/dl, with a standard deviation of 2.95 g/dl, which is within the normal range. Additionally, the mean baseline fasting blood sugar (BLFBS) level was 172.98 mg/dl, with a standard deviation of 11.81 mg/dl. This elevated mean BLFBS level suggests that most patients were hyperglycemic at the start of treatment, as it exceeds the normal fasting blood sugar threshold of less than 126 mg/dl Table 1.

Table 2 presents the median time required for diabetes mellitus patients to achieve optimal glycemic control. The median time to achieve glycemic control is estimated to be 12 months, with a standard error of 0.482 months. The 95% confidence interval for the median time ranges from 11.06 to 12.95 months, suggesting that half of the patients achieved glycemic control within 12 months, while the other half took longer.

**Table 1:** Summary statistics for predictor variables by censored and event status.

Variable	Category	Censored (%)	Event (%)	Total (%)
Gender	Male	78 (18.3)	126 (29.5)	204 (47.8)
	Female	32 (7.5)	191 (44.7)	223 (52.2)
Residence	Rural	48 (11.2)	187 (43.8)	235 (55.0)
	Urban	62 (14.5)	130 (30.4)	192 (45.0)
Presence of HTN	No	88 (20.6)	247 (57.8)	335 (78.5)
	Yes	22 (5.2)	70 (16.4)	92 (21.5)
Presence of CKD	No	37 (8.7)	367 (86.0)	404 (94.6)
	Yes	14 (3.3)	9 (2.1)	23 (5.4)
FHDM	No	74 (17.3)	195 (45.7)	269 (63.0)
	Yes	36 (8.4)	122 (28.6)	158 (37.0)
History of DM related	No	79 (18.5)	253 (59.3)	332 (77.8)
Complication	Yes	31 (7.3)	64 (15.0)	95 (22.2)
Comorbidity	No	67 (15.7)	237 (55.5)	304 (71.2)
	Yes	43 (10.1)	80 (18.7)	123 (28.8)
	Mean	SD		
Baseline measured continuous covariates				
Weight	58.26	4.93		
Age	43.85	3.74		
Hemoglobin level	12.76	2.95		
BLFBS	172.98	11.81		

**Table 2:** Median time to optimal glycaemic control.

Median for survival time			
Median estimate	SE	95% confidence interval	
12	0.482	Lower bound	Upper bound
		11.055	12.945

### Non parametric analysis for survival data

**Fig. 2:**
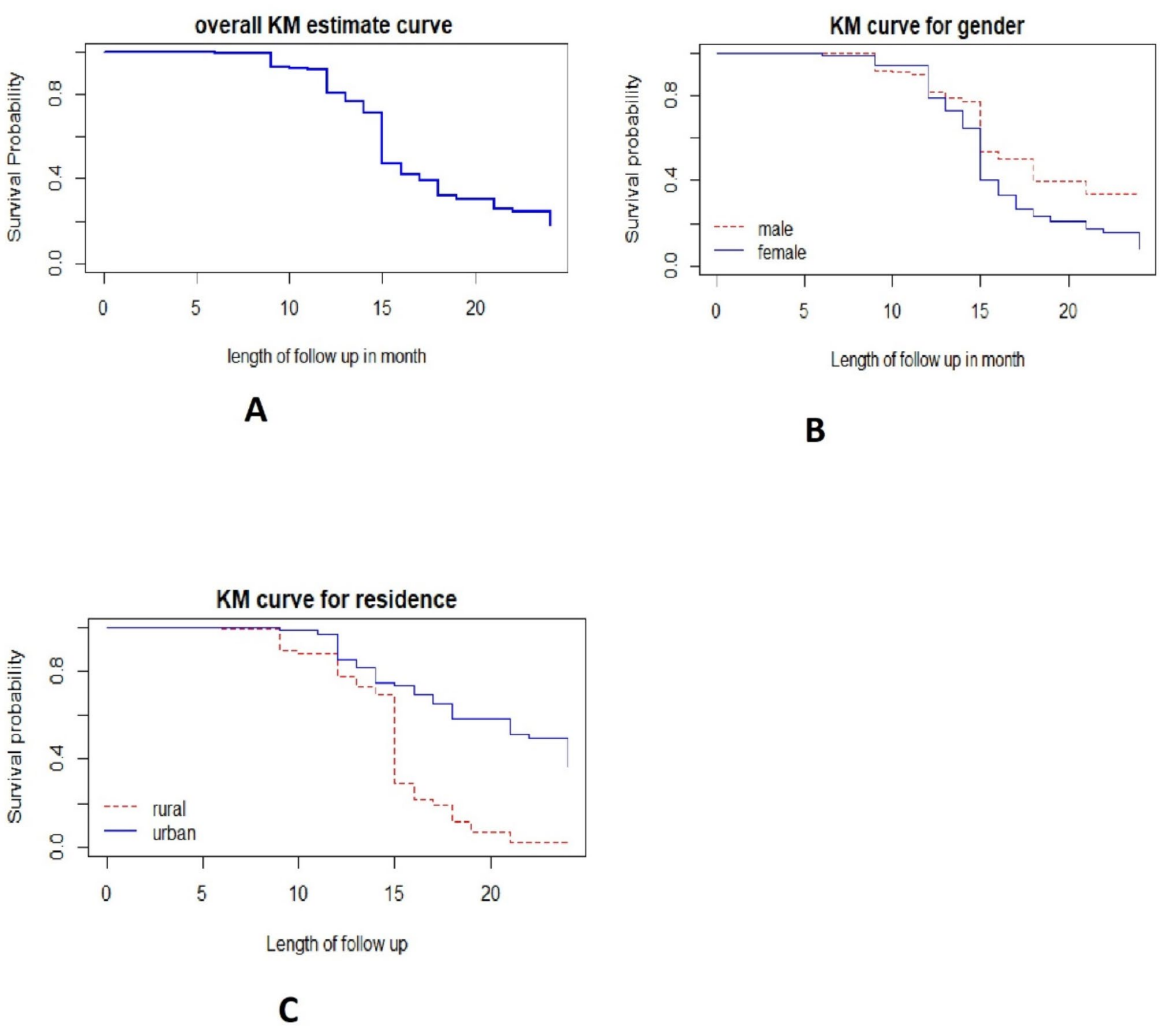
Kaplan–Meier survival estimate curves.

A Kaplan-Meier estimator was used to estimate the survival time. The overall Kaplan-Meier survival curve in (Fig. 2A) showed that as time increased, the curve decreased rapidly, indicating that most patients recovered from diabetes mellitus. This means that as time progressed, the waiting time of patients with T1DM decreased monotonically.

Separate Kaplan-Meier survival curves were constructed to estimate survival time based on different covariates, allowing us to examine differences in recovery rates between categories of individual covariates. From the Kaplan-Meier survival curves of individual covariates:

The plot in Fig. 2B suggested that the time to achieve optimal glycemic control was longer for male patients compared to female patients. This indicates that male patients had a longer recovery time than females. Similarly, the plot in Fig. 2C suggested that the time to achieve optimal glycemic control was longer for urban residents compared to patients from rural areas. In other words, urban patients took longer to reach optimal glycemic control than rural patients. The remaining variables were described in a similar manner.

### Log rank test

The log-rank test was performed at a 5% level of significance to compare the survival experiences across different categories of all categorical predictors. There were significant differences in recovery rates between males and females, patients with and without related comorbidities, urban and rural patients, patients with and without hypertension, patients with a history of DM complications and those without, as well as patients with and without a family history of DM Table 3.

**Table 3:** Log rank tests for categorical variables.

Covariates	DF	Chi-square	*P*-value
Gender	1	15.09	< 0.001
Residence	1	28.91	< 0.0001
Presence of comorbidity	1	8.6	0.0033
FHDM	1	5.4	0.02
Hypertension	1	7.47	0.006
CKD	1	3.52	0.051
History of DM complication	1	18.09	< 0.0001

### Multivariable analysis for Cox proportional hazard model

The results of the multivariate Cox Proportional Hazards (Cox-PH) model indicate that several factors significantly influence the time to achieve optimal glycemic control. Age was found to have a significant negative association with the likelihood of achieving optimal glycemic control, with an adjusted hazard ratio (AHR = 0.871(95% CI 0.809, 0.937)). This implies that for each additional year of age, the likelihood of achieving optimal glycemic control decreases by approximately 13%. Gender was also significantly associated with glycemic control, where female patients were 29.5% more likely to achieve glycemic control compared to their male counterparts (AHR = 1.295 (95% CI 1.024, 1.639)).

Patients with a FHDM were 68.1 times more likely to achieve optimal glycemic control compared to those without a FHDM (AHR = 1.681 (95% CI 1.313, 2.153)). Residence was another significant factor; urban patients were 53.7% less likely to achieve glycemic control compared to rural patients (AHR = 0.463 (95% CI 0.354, 0.607)). The presence of comorbidities was associated with a reduced likelihood of achieving optimal glycemic control, with patients who had related comorbidities being about 49.2% less likely to achieve glycemic control compared to those without related diseases (AHR = 0.508 (95% CI 0.302, 0.854)). Additionally, The presence of DM-related complication was associated with a reduced likelihood of achieving optimal glycemic control, with patients who had DM-related complications being about 58.1% less likely to achieve glycemic control compared to those without related comorbidities (AHR = 0.419 (95% CI 0.326, 0.539)). Baseline fasting blood sugar was also found to be a significant determinant, with an (AHR = 0.997 (95% CI 0.995, 0.998)), indicating that higher baseline blood sugar levels are associated with a slight delay in achieving optimal glycemic control.

In summary, younger age, female gender, family history of diabetes mellitus, and rural residence were positively associated with a shorter time to achieve glycemic control. Conversely, the presence of comorbidities, DM complications, and higher baseline fasting blood sugar levels were associated with a prolonged time to achieve glycemic control. Hemoglobin levels showed a non-significant trend toward decreasing the likelihood of achieving glycemic control. These findings provide critical insights into the factors affecting the time to achieve glycemic control, highlighting areas for targeted interventions to improve diabetes care outcomes (Table 4).

**Table 4:** The multivariate Cox proportional hazards model analysis.

Covariate	Category	AHR (95% CI)	*P*-value
Age		0.871 (0.809, 0.937)	< 0.0001***
Gender	Male	1	
	Female	1.295 (1.024, 1.639)	0.031*
Residence	Rural	1	
	Urban	0.463 (0.354, 0.607)	< 0.0001***
Presence of HTN	No	1	
	Yes	0.416 (0.122, 1.423)	0.162
Comorbidity	No	1	
	Yes	0.508 (0.302, 0.854)	0.011*
FHDM	No	1	
	Yes	1.681 (1.313, 2.153)	< 0.0001***
DM related complication	No		
	Yes	0.419 (0.326, 0.539)	< 0.0001***
Hemoglobin		0.989 (0.973, 1.006)	0.224
BLBGL		0.997 (0.995, 0.998)	< 0.0001***

### Model diagnostics

In any statistical analysis, after the model fitting process, it is essential to check the model assumptions. The Schoenfeld residuals and Cox-Snell residuals were used to assess the proportional hazards assumption of the survival analysis.

**Fig. 3:**
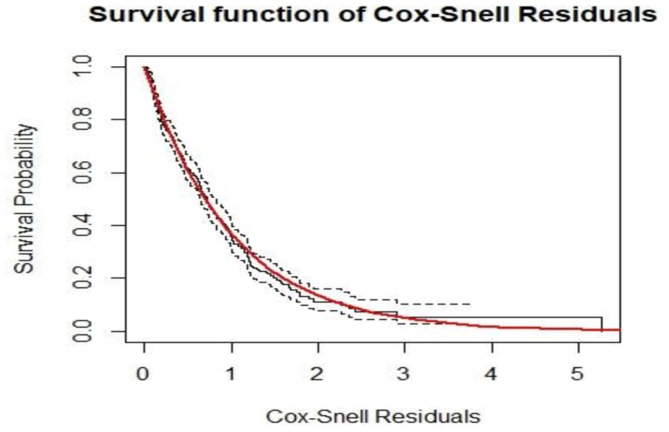
Cox-snell residual plots for time to optimal glycemic control.

We checked the survival model assumption graphically using Cox-Snell residual plots. In Fig. 3, the diagnostic plot based on Cox-Snell residuals, with the 95% point-wise confidence interval (CI) for the Kaplan-Meier estimate of the Cox-Snell residuals, is shown along the red line. The survival function of the unit exponential distribution indicates that the survival function of the standard exponential distribution lies within the 95% CI of the Kaplan-Meier estimate. This suggests that the survival process model fits the data well.

However, graphical tests alone are not sufficient to be certain about the proportional hazards assumption of the model because they are subject to interpretation and can vary between individuals. Therefore, in addition to graphical tests, it is recommended to use statistical tests. To test this assumption, the global test of Schoenfeld residuals was used. Based on the results in Table 5, the p-value for the global test was not significant, and the p-values for each predictor variable were also insignificant. Hence, the proportional hazards model assumption was not violated.

**Table 5:** Cox proportional hazard assumption test.

Variable	Ch-square	Df	*P*-value
Age	0.965	1	0.325
Gender	3.941	1	0.047
FHDM	2.793	1	0.094
Comorbidity	1.461	1	0.226
HTN	2.168	1	0.141
DM related complication	1.221	1	0.269
Hemoglobin	0.396	1	0.529
Residence	0.820	1	0.365
BLBGL	1.161	1	0.281
Global	15.417	9	0.080

## Discussion

The main objective of this study was to identify the determinant factors of the time to optimal glycemic control for T2DM patients under treatment at Assosa General Hospital, Assosa, Western Ethiopia. In the analysis of this survival data, the non-parametric survival analysis techniques (Kaplan-Meier and log-rank test) were used for comparing the survival experience of categorical predictor variables. Also, the semi-parametric (Cox proportional hazard regression) analysis was used to investigate the determinant factors of time to optimal glycemic control for T2DM patients.

Age was found to have a significant negative association with the likelihood of achieving optimal glycemic control, implies that for each additional year of age, the likelihood of achieving optimal glycemic control decreases by approximately 13%. This result is consistent with another study^24,40,41^. In their findings, Targets for glycaemic control in older adults with T2DM are often less stringent than in younger adults to avoid hypoglycaemia and minimize unbeneficial interventions.

Baseline fasting blood sugar (BLFBS) was also a statistically significant variable. As the BLFBS level increases, the likelihood of patients reaching optimal glycemic control decreases. This result is consistent with another study^42,43^. In their findings, higher baseline HbA1c levels reduced the likelihood of achieving early glycemic control (adjusted relative risks were lower for those with high baseline levels. Similarly, this study aligns with the findings of^44^, where it was noted that monitoring and lowering blood sugar provides an effective approach to controlling diabetes mellitus.

Also, Patients who had other related comorbidity prolonged the time to reach glycemic control. This result was consistent with another study^23,45^. In their finding, in patients who have related diabetic complications and other diseases like HIV/AIDS, kidney disease, stroke, hypertension, and the like; the tendency to control their glycemic level is low. That is diabetic patients who also have another related disease may affect the first recovery time of patients because of the additional disease they have.

Rural patients achieved optimal glycemic control in a shorter time compared to urban patients. This result aligns with another study^24^. However, it contradicts the findings of a different study^27,46^, which reported that urban patients were better able to control and reduce their blood sugar over time. The observed discrepancy in recovery times between rural and urban diabetes mellitus patients may be attributed to several factors. While urban areas provide better healthcare access, rural patients may benefit from more personalized follow-up care. Additionally, rural patients often engage in more physical activity and consume unprocessed foods, which enhance glycemic control. In contrast, urban lifestyles tend to be more sedentary, with greater exposure to processed foods. Higher stress levels in urban environments may further hinder recovery. Socioeconomic disparities and differences in health literacy also contribute, as urban areas, despite having more resources, face inequalities that can affect outcomes. Variations in study design, including sample size and population characteristics, may also explain the conflicting findings. This highlights the need for further research to better understand the impact of rural versus urban living on diabetes management.

Female patients reached glycemic control in a shorter time compared to male patients. This result aligns with the findings of studies^24,42^, which reported that females tend to recover faster than males. However, it contradicts another study^47^, which revealed that after one year of treatment, 38.9% of women and 40.6% of men achieved the target HbA1c level, indicating a slight but significant difference favoring men. The differences in glycemic control between male and female patients may be due to several factors, including biological, behavioral, and psychosocial influences, sample-specific factors, geographical differences, and measurement and study design differences. Females may achieve better glycemic control due to higher health-seeking behavior, better adherence to treatment, and greater awareness of health conditions. They are also more likely to follow dietary recommendations and attend regular follow-up visits, which can contribute to faster recovery. On the other hand, studies showing that males have a higher probability of achieving glycemic control may reflect biological differences, such as hormonal variations that can influence insulin sensitivity. In addition, males might have fewer psychosocial barriers, such as caregiving responsibilities, that can interfere with self-care and treatment adherence. Variations in the study populations, such as differences in sample size, age distribution, comorbidities, and socioeconomic status, could further explain the conflicting findings. Differences in healthcare access, cultural norms, and study methodologies may also contribute to the observed contradiction. This highlights the need for more context-specific research to explore gender differences in diabetes management outcomes.

Finally, patients with a family history of diabetes mellitus recovered more quickly. This result is aligns with^48^, which found that having a family member with T2D facilitated self-management in several ways. Personal familiarity with T2D and related lifestyle changes, as well as advice from older relatives, led individuals to feel more capable of modifying behaviors and made adjusting to the diagnosis easier. Similarly, another study indicated that a family history of diabetes is linked to better awareness of risk factors, increased fruit and vegetable consumption, and higher participation in diabetes screening, reflecting a proactive approach to disease management^49^. However, this finding contradicts the results of studies^27,50^, which suggested that patients with no family history of diabetes mellitus are better able to monitor their sugar levels. This discrepancy requires further investigation. The discrepancy could be due to differences in patient perceptions, motivations, and behaviors between those with and without a family history of diabetes. Patients with a family history may have a deeper understanding of the importance of managing diabetes, which could lead them to engage in more proactive health behaviors and thus recover more quickly. On the other hand, patients without a family history may be more vigilant about monitoring their sugar levels because they have no prior exposure to the disease and are more cautious about preventing it. Additionally, it is possible that patients without a family history may have more access to resources or healthcare, which could help them manage their condition more effectively. Differences in study design, sample populations, and healthcare settings could also contribute to the contradictory results.

## Limitations

This study was done by a well-defined statistical model and this should give a more appropriate result. Since there were scarce of studies about non-communicable disease like diabetes mellitus done in Western Ethiopia, this finding was used as input for other studies who wanted to do research in this area or anywhere. Our findings were also subject to some limitations. This study has several limitations. The retrospective design may introduce potential biases, such as inaccuracies or inconsistencies in medical record documentation, which could affect the reliability of the data. Additionally, the observational nature of the study limits the ability to infer causal relationships between the time to optimal glycemic control and its associated factors. The other limitation is that the AHR for variables did not exceed twice the reference group values, which may limit the strength of association. Future prospective studies with rigorous data collection methods are recommended to validate these findings and further explore the determinant factors of glycemic control.

## Conclusion

In general, this study identified factors that either prolonged or shortened the time required to achieve optimal glycemic control in T2DM patients. The findings indicated that older age, male gender, the presence of comorbid conditions, absence of FHDM, and diabetes-related complications were poor prognostic factors, contributing to prolonged recovery time. Therefore, special attention should be given to these patient groups to help them achieve better glycemic control and overall health.

## Data Availability

The datasets generated and/or analyzed during the current study are not publicly available due to ethical concerns, confidentiality agreements, or legal restrictions. However, the data can be obtained by contacting the corresponding author of the study and making a reasonable request for access to the data.

## Abbreviations

AGH: Assosa General Hospital
DM: Diabetes mellitus
T1DM: Type 1 diabetes mellitus
T2DM: Type 2 diabetes mellitus
FHDM: Family history of diabetes mellitus
Mg/dl: Milligram per deciliter
OPD: Outpatient department
BLBGL: Baseline blood glucose level
